# P-1325. Quality of Life and Severity of Symptoms in 1-year Follow Up of Patients Presenting with Acute Babesiosis: A Prospective Cohort Study with Validated Questionnaire

**DOI:** 10.1093/ofid/ofae631.1503

**Published:** 2025-01-29

**Authors:** Rudline G Zamor, Brigitte Maczaj, Sarath Nath, Aikaterini Papamanoli, Bennadette Maramara, Michael D Lum, Xiaoyue Zhang, Jie Yang, Eric Spitzer, Dana Mordue, Luis A Marcos

**Affiliations:** Stony Brook University Hospital, Stony Brook, New York; Stony Brook Medicine, Stony Brook, New York; Stony Brook University Hospital, Stony Brook, New York; Stony Brook University Hospital, Stony Brook, New York; Stony Brook University Hospital, Stony Brook, New York; Stony Brook University Hospital, Stony Brook, New York; Stony Brook Medicine, Stony Brook, New York; Stony Brook Medicine, Stony Brook, New York; Stony Brook Medicine, Stony Brook, New York; New York Medical College, Valhalla, New York; Renaissance School of Medicine at Stony Brook University, Stony Brook, New York

## Abstract

**Background:**

Tick-borne diseases are a growing public health concern in the US, particularly in New York. *Babesia microti*, a parasitic blood-borne piroplasm, causes the disease Babesiosis in humans. The aim of this prospective cohort study is to examine the frequency and severity of symptoms as well as the Quality of Life (QOL) after treatment in patients with Babesiosis (immunocompetent vs. immunocompromised) at up to 1-year of follow up.

**Table 1:**
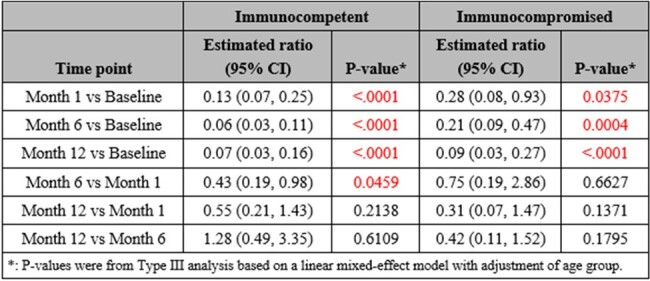
Estimated ratio of VAS total score across time points within each immune group based on linear mixed effect model

**Methods:**

Acute babesiosis adult patients with peripheral blood smear positive for Babesia (confirmed *B. microti* by PCR) were enrolled into a 1-year follow up cohort study. Twelve symptoms were evaluated by a Visual Analogue Scale (VAS) scoring system on presentation, and at 1, 6 and 12 months; higher scores represented worse status. QOL was assessed through a 36-Item Short Form Survey (SF-36) which taps eight health concepts; higher scores represented better status. Linear mixed-effects models were constructed to examine the change in VAS and SF-36 scores.

**Table 2:**
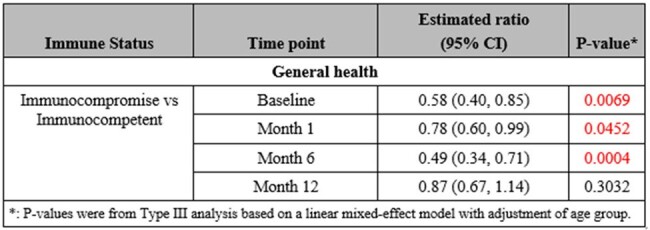
Estimated ratio of SF-36 concept score between immune groups at each time point based on linear mixed effect models

**Results:**

Out of a total of 38 enrolled, 33 patients (mean age 60 ± 19 years; female 30.3%) completed their initial VAS. At baseline, fatigue had the highest frequency among all symptoms, 96.15% and 100%, in the immunocompetent and immunocompromised cohorts, respectively. Median VAS scores at baseline were 35.5 (immunocompetent, n=26) and 22.0 (immunocompromised, n=7). Compared to baseline, VAS total symptom scores significantly decreased at 1 month for the immunocompetent (n=20, p < .0001) and immunocompromised groups (n=5, p=0.04). For SF-36 concepts, 29 patients completed the initial survey (immunocompromised, n=6). Within QOL, concept scores for physical functioning and general health were significantly increased at 6 months follow up in both cohorts. Nonetheless, in comparison to the immunocompetent group, the immunocompromised group had significantly lower scores, or worsened QOL, in general health concept at months 1 (p=0.04) and 6 (p=0.0004).

**Conclusion:**

VAS scores significantly decreased for both groups, but more prominently for the immunocompetent group, and a difference in quality of life was identified. Immunocompromised patients had more prolonged symptoms and worsened quality of life during the 1-year follow up compared to healthy individuals.

**Disclosures:**

**All Authors**: No reported disclosures

